# Functional Characterization of the *alb1* Orthologue Gene in the Ochratoxigenic Fungus *Aspergillus carbonarius* (*AC49* strain)

**DOI:** 10.3390/toxins10030120

**Published:** 2018-03-12

**Authors:** Donato Gerin, Luis González-Candelas, Ana-Rosa Ballester, Stefania Pollastro, Rita Milvia De Miccolis Angelini, Francesco Faretra

**Affiliations:** 1 Department of Soil, Plant and Food Sciences, University of Bari Aldo Moro, via Amendola 165/A, 70126 Bari, Italy; donato.gerin@uniba.it (D.G.); ritamilvia.demiccolisangelini@uniba.it (R.M.D.M.A.); francesco.faretra@uniba.it (F.F.); 2 Instituto de Agroquímica y Tecnología de Alimentos, IATA-CSIC, Calle Agustín Escardino 7, Paterna, 46980 Valencia, Spain; lgonzalez@iata.csic.es (L.G.-C.); ballesterar@iata.csic.es (A.-R.B.); 3 SELGE Network of Public Research Laboratories, via Amendola 165/A, 70126 Bari, Italy

**Keywords:** melanin, *alb1*, OTA partitioning, gene disruption, conidiation, sclerotia

## Abstract

*Aspergillus carbonarius,* belonging to the group *Nigri*, is the main species responsible for contamination by ochratoxin A (OTA) in grapes and derivative products. OTA can accumulate in the mycelium and in black conidia of the fungus and released into the matrix. Here, we have deleted in *A. carbonarius* the *alb1* orthologue gene of *A. fumigatus*, involved in melanin biosynthesis. Three *A. carbonarius* Δ*alb1* mutants were characterized for morphologic traits and OTA production on different media and temperatures. Δ*alb1* mutants showed a fawn color of conidia associated with a significant reduction of the conidiogenesis and a statistically significant increase (*p* ≤ 0.01) of total OTA production as compared to the wild type (WT) strain. The *alb1* gene somehow affected OTA partitioning since in Δ*alb1* mutants OTA amount was lower in conidia and was more abundantly secreted into the medium as compared to the WT. On grape berries the Δ*alb1* mutants and the WT caused lesions with similar sizes but OTA amount in berry tissues was higher for the mutants. These results demonstrate that *A. carbonarius* conidia pigmentation is largely dependent on polyketide biosynthesis. The gene is not directly involved in virulence and its deletion affects morphological features and OTA production in the fungus.

## 1. Introduction

*Aspergillus carbonarius*, belonging to the section *Nigri* (black Aspergilli), is the main species responsible for the contamination by ochratoxin A (OTA) in grapes and derivatives [1,2,3]. OTA is a mycotoxin with potent nephrotoxic and immunosuppressive effects and it has been classified as a possible human carcinogen (group 2B) [4].

A common feature of all *Aspergillus* species belonging to section *Nigri*, including *A. carbonarius*, is the dark pigmentation of conidia, probably due to the synthesis of aspergillin resulting from the combination of the green pigment hexahydroxyl pentacyclic quinoid and the brown pigment melanin [5]. Several studies focused on melanin in *Aspergillus*, but most information on this issue comes from *A. fumigatus* and several aspects, such as its precise location, amount, type, and physico-chemical nature remain to be clarified [6]. For *A. carbonarius*, in particular, Babitskaya et al. [7] proved that the pigments synthesized by the fungus are melanins of the dihydronaphthalene type.

The genetic basis of conidia pigmentation has been fully elucidated in *A. fumigatus*, in which it is due to the synthesis of 1,8-dihydroxynaphtalene (1,8-DHN) melanin [8]. A cluster of six genes was identified to be involved in the biosynthetic pathway to produce 1,8-DHN melanin and inactivation of single genes in the cluster caused different pigmentation of conidia [9]. The cluster contains the genes *alb1* (synonymous of *pksP*), *ayg1*, *arp2*, *arp1*, *abr1*, *abr2* encoding, in the order, a polyketide synthase (PKS) involved in the production of the heptaketide naphtopyrone YWA1 [10], a putative hydrolase converting YWA1 into 1,3,6,8-tetrahydroxynaphtalene (1,3,6,8-THN) [11], an hydroxynaphthalene (HN) reductase yielding both scytalone and vermelone [8], a dehydratase converting scytalone in 1,3,8-THN [8], a putative multicopper oxidase converting vermelone to 1,8-DHN [12] and, finally, a laccase that polymerize 1,8-DHN in 1,8-DHN melanin [13]. In contrast, in *A. niger*, the genes *fwnA*, *olvA* and *brnA* (orthologues of *alb1*/*pksP*, *ayg1* and *abr1*, respectively) are involved in the production of the characteristic black pigment of conidia but are not organized in a cluster [14].

PKS are multi-domain enzymes playing a key role in fungal secondary metabolism [15]. Recently, a RNA-Seq study using four *A. carbonarius* strains showed that different *pks* were up-regulated under OTA-inducing conditions. In addition to the *pks* gene involved in OTA production (ID: 173482; DOE Joint Genome Institute; http://www.jgi.doe.gov), the most up-regulated *pks* gene (ID: 172075) under the tested conditions corresponded to the orthologue of *alb1*/*fwnA* genes of *A. fumigatus/A. niger*, not included in a gene cluster [16].

In the present study, *Agrobacterium tumefaciens*-mediated transformation (ATMT) was used for gene disruption of the *pks* gene (ID: 172075) in *A. carbonarius*. Three Δ*alb1* strains were characterized for phenotypic features, including morphological traits, OTA production in vitro and in vivo and its partitioning in conidia, mycelium and medium in comparison to the wild-type strain.

## 2. Results

### 2.1. A. carbonarius alb1 Gene Description and Phylogenetic Analysis

**Figure 1:**
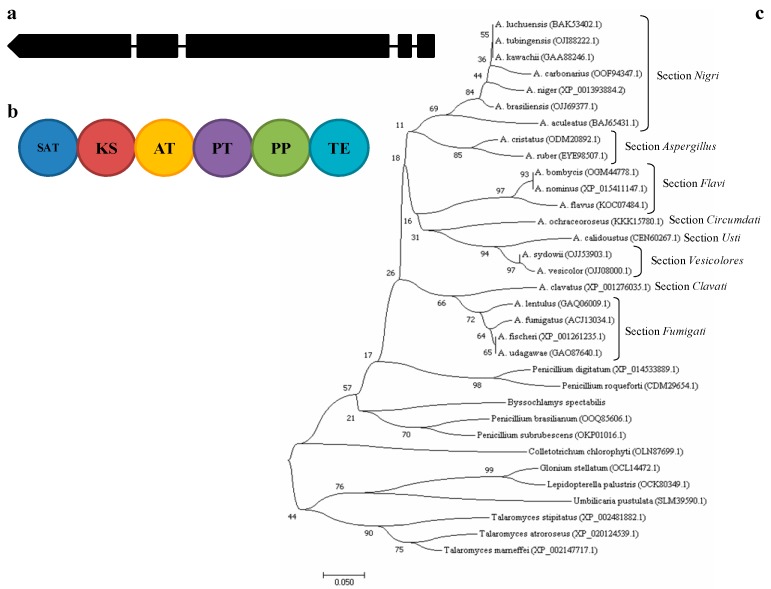
Gene structure (**a**) and domains structure (**b**) of *alb1*(ALB1) in *A. carbonarius*, and phylogenetic analysis (**c**) of ALB1 AT domain in different fungal species. SAT: ACP-transcyclase; KS: β-ketoacyl synthase; AT: acyl transferase; PT: product template; PP: phosphopantetheine attachment site; TE: thioesterase. The tree was constructed by the neighbor-joining method using Poisson correction. Bootstrap values were calculated from 1000 trees. The sequence accession number for each species is reported in parenthesis.

The *pks* ID: 172075 of *A. carbonarius* is the orthologue gene of the deeply studied *alb1* gene of *A. fumigatus* (identity 72%) and the *fwnA* gene of *A. niger* (identity 89%) involved in the first step of DHN-melanin biosynthesis. It consists of five exons separated by short (48–62 bp) introns and encodes for a type I PKS, composed by the domains ACP-transcyclase (SAT), β-ketoacyl synthase (KS), acyl transferase (AT), product template (PT), phosphopantetheine attachment site (PP) and thioesterase (TE) (Figure 1a,b).

The gene seems to be conserved in the ascomycetes (Table S1) and according to phylogenetic analysis of the AT domain, the sequence is generally clade based on sections and species within the genus *Aspergillus*, in fact the highest bootstrap values include species having similar conidia pigmentation (Figure 1c).

### 2.2. Generation of A. carbonarius Δalb1 Mutants

**Figure 2:**
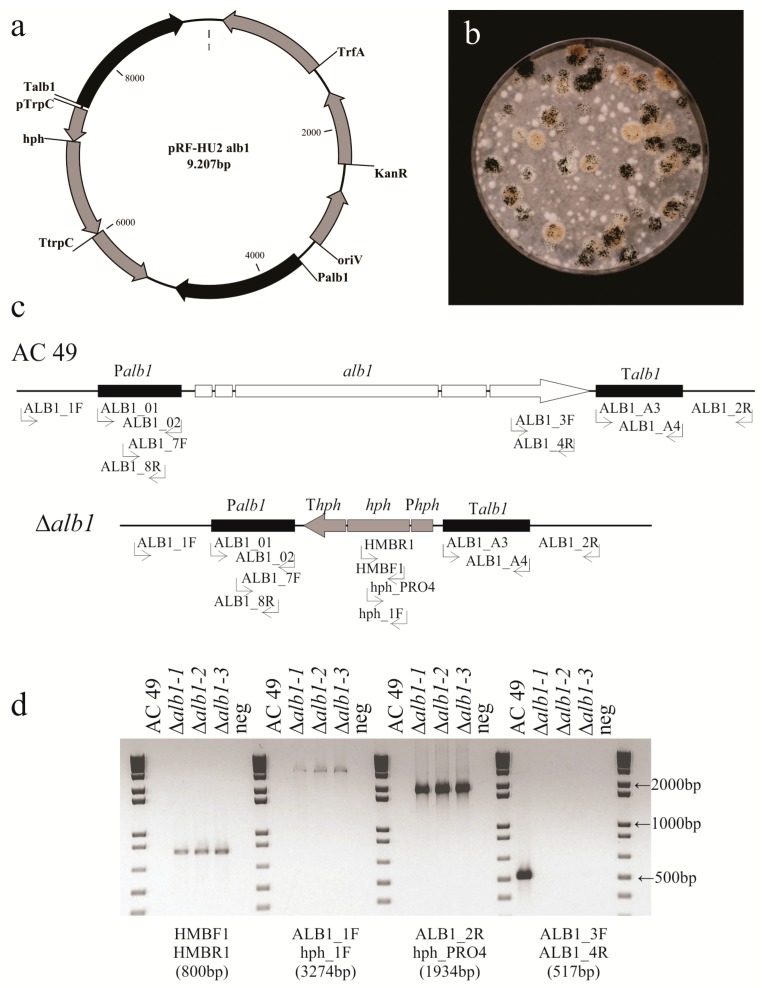
Analysis of *Aspergillus carbonarius alb1* transformants. (**a**) Map of plasmid pRF-HU2_alb1. (**b**) Transformation plate containing colonies displaying fawn pigmented conidia (putative Δ*alb1*) or wild-type-like morphology regarding conidia pigmentation. (**c**) Diagram of the wild-type locus and the *alb1* replacement with the hygromycin resistant selectable marker from pRF-HU2_alb1 by homologous recombination to generate the Δ*alb1* mutants. Primers used in the construction of plasmid pRF-HU2_alb1 and those used for the analysis of the transformants are shown. (**d**) Polymerase chain reaction (PCR) analysis of the wild-type *AC49* strain and three knockout transformants (Δ*alb1-1*, Δ*alb1-2*, Δ*alb1-3*).

To investigate whether the *alb1* gene is involved in the biosynthesis of melanin and in the production of OTA, the gene was deleted in the *A. carbonarius* *AC49* strain by replacing it with a hygromycin resistance cassette. The gene replacement binary plasmid pRF-HU2alb1 was constructed with the 1.4 kb upstream and downstream fragments of the *alb1* gene, which were cloned in the vector pRF-HU2 [17] flanking the hygromycin resistance marker. The resulting plasmid (Figure 2a) was used to obtain the *A. carbonarius alb1* knockout mutants (Δ*alb1*) via *A. tumefaciens* mediated transformation (ATMT). One hundred and six hygromycin resistant colonies were obtained in co-cultivation of 1.5 × 10^4^ conidia/mL with *A. tumefaciens*, corresponding to a transformation frequency of 0.7%. About 70% of the colonies yielded fawn pigmented conidia while the remaining colonies displayed wild-type morphologic traits (Figure 2b). Thirty fawn-colored colonies were selected for subsequent analysis. To confirm the presence of the hygromycin gene we used the primers HMBF1 and HMBR1 (Table 1 and Figure 2c). The expected 800-bp PCR fragment was detected in all transformants and was absent in the untransformed *AC49* strain (Figure 2d). Confirmation of the deletion of the target gene was obtained using primers that flanked either the 5′ or 3′ end of the construct in combination with primers within the hygromycin resistant marker (ALB1_1F and hph_1F for the 5′ part, and ALB1_2R and hph_PRO4 for the 3′ part, Figure 2c), as only homologous recombinants (deletants) would amplify with these two primer pairs (Figure 2d). Monosporic isolates were obtained from these transformants and were further validated by PCR using primers located within the coding regions (ALB1-3F and ALB1-4R, Figure 2c), which is not present in the T-DNA. As expected, these primers amplified in the wild-type, but failed to amplify the knockout Δ*alb1* mutants (Figure 2d).

Six knockout transformants were selected for determination of the number of T-DNA copies integrated in the genome by quantitative PCR using the wild-type *AC49* strain as a control and the *nrps* gene as a reference (Table 1). The six Δ*alb1* mutants analyzed contained a single T-DNA integration, so we selected three of them (Δ*alb1-1*, Δ*alb1-2*, and Δ*alb1-3*) for further functional characterization of the *alb1* gene (Table S1).

### 2.3. Phenotypic Characterization

**Figure 3:**
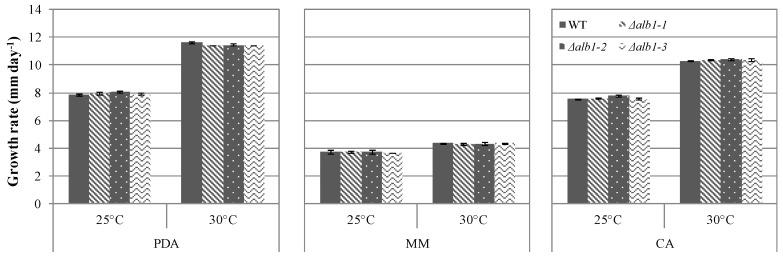
Growth rate at 25 °C and 30 °C of the Δ*alb1* mutants and the WT strain on different media. Figures are the mean values of three technical replicates. No statistical differences were observed among strains through three-way ANOVA analysis.

No statistical differences in the daily colony growth were observed among Δ*alb1* and WT strains (Figure 3). The colony growth was medium-dependent and on all the tested medium the strains grew faster at 30 °C than at 25 °C. On MM the daily growth rate was reduced of 50–62% as compared to CA and PDA (Figure 3).

**Figure 4:**
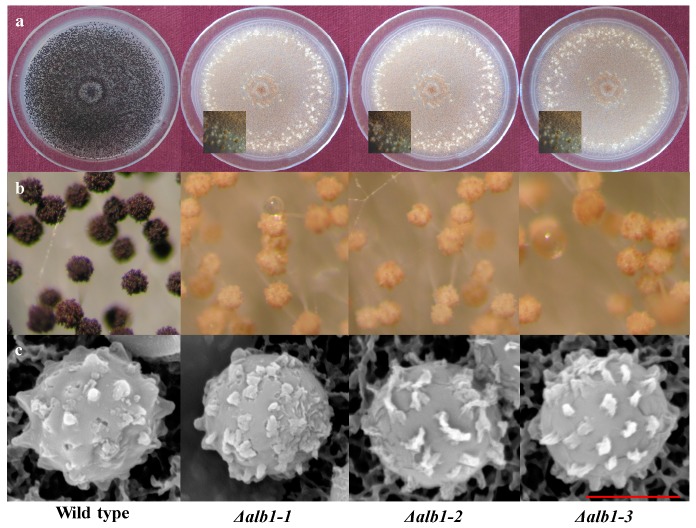
Morphology of 7-day-old colonies grown on PDA of the *A. carbonarius* Δ*alb1* mutants and the WT strain. (**a**) Colony with detail of sclerotia produced by the Δ*alb1* mutants; (**b**) Conidiophores observed by stereomicroscopy; (**c**) SEM study of conidial surface (Scale bar: 5 μm).

**Figure 5:**
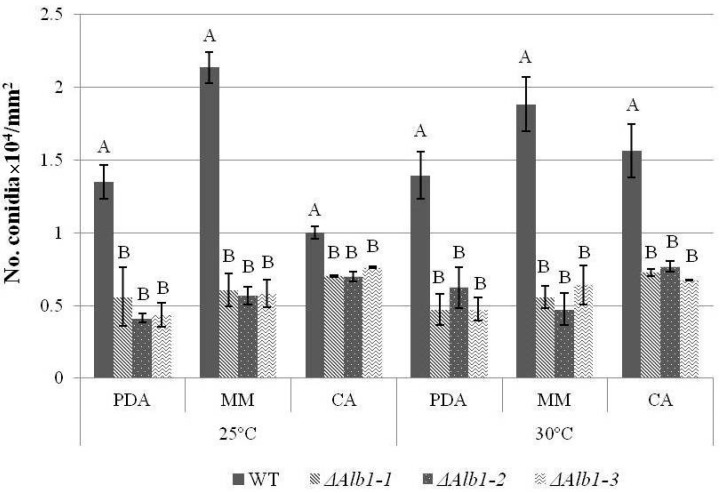
Production of conidia in the Δ*alb1* mutants and the WT strain. Figures are the mean values of three technical replicates. Values followed by the same letter within the same medium for each temperature are not statistically different (Tukey’s test at probability level *p* ≤ 0.01).

Substantial changes occurred in asexual sporulation. Conidia produced by Δ*alb1* mutants showed a fawn pigmentation as opposed to the typical black pigmentation of WT conidia (Figure 4a). Stereomicroscope observations showed that the conidiophore vesicles of Δ*alb1* mutants carried a lower number of conidial chains as compared to the WT strain (Figure 4b). The numbers of conidia produced at 7 DAI by Δ*alb1* mutants were significantly (*p* ≤ 0.01) lower than those produced by the WT strain in all the tested conditions (Figure 5), whereas temperature and media did not significantly influence the conidiogenesis (Table S2). In detail, the WT strain produced from 1.0 × 10^4^ (CA, 25 °C) to 2.1 × 10^4^ conidia/mm^2^ (MM, 25 °C), and the Δ*alb1* mutants produced from 0.4 × 10^4^ (Δ*alb1-2*, PDA, 25 °C) to 0.8 × 10^4^ conidia/mm^2^ (Δ*alb1-3*, CA, 25 °C). The morphology of the conidial surface was investigated through SEM observations and similar echinulate surfaces were observed in WT and Δ*alb1* strains (Figure 4c).

In Δ*alb1* mutants the production of sclerotia was promoted as compared to WT on all the media (PDA, MM and CA) and at both temperatures (25 °C and 30 °C) (Table 2). After 7 DAI the sclerotia at 25 °C ranged from 4 to 11 on MM, from 9 to 23 on PDA and from 44 to 56 on CA. The number of sclerotia remarkably increased at 30 °C on MM (25 to 26) and PDA (81 to 108) and much less on CA (49 to 66). No differences in sclerotial size were recorded and sclerotia were 800–1300 μm in diameter, in agreement with previously reported findings [18].

### 2.4. OTA Production and Partitioning

Evaluation on total OTA showed that Δ*alb1* mutants produced more toxin than the WT strain (Table 3) and that it was significantly (*p* ≤ 0.01) influenced by temperature and media (Table S3). Indeed, a higher amount of OTA was produced at 25 °C than at 30 °C, and it was more abundant on CA followed by PDA and MM. However, the biggest differences in OTA production between Δ*alb1* mutants and the WT strain was recorded on MM (227.2 to 755.9%), followed by CA (66.3 to 168.9%) and PDA (32.5 to 70.8%) (Table 3).

The OTA partitioning in conidia, mycelium and medium was assessed at different DAI in the Δ*alb1* mutants and the WT strain grown on CA medium. Generally, the higher amount of OTA in the mycelium was quantified at 4 DAI with no differences between Δ*alb1* and WT (Table 4). In the WT strain the OTA rapidly decreased from 3.3 ng mg^−1^ (4 DAI) to 0.4 ng mg^−1^ (6 DAI), and then lightly increased to 1.0 ng mg^−1^ (8–10 DAI). The same behavior was observed for the Δ*alb1* mutants, even if a greater amount of OTA was quantified at 6 (0.9–1.6 ng mg^−1^), 8 (1.3–2.9 ng mg^−1^) and 10 (1.9–3.3 ng mg^−1^) DAI as compared to WT. OTA concentrations were always higher in WT conidia (3.0–23.5 pg 10^−2^ conidia) than in conidia of the Δ*alb1* mutants (1.2–12.1 pg 10^−2^ conidia) and statistically significant differences (*p* ≤ 0.05) were observed at 6 and 10 DAI. In contrast to this, Δ*alb1* mutants secreted more OTA than WT in the medium (6.6 to 13.9 and 3.8 to 9.7 ng mg^−1^, respectively) and differences were statistically significant (*p* ≤ 0.05) only at 4 and 6 DAI (Table 4).

In Δ*alb1* mutants, OTA was accumulated also in the sclerotia, and when they were differentiated concentrations ranged from 29.8 ng mg^−1^ to 1378.1 ng mg^−1^.

### 2.5. Artificial Inoculation on Grape Berries

The behavior of Δ*alb1* mutants on artificially-inoculated grape berries was evaluated at 7 DAI (Figure S1). Generally, the lesions caused by the Δ*alb1* mutants were bigger in size than those caused by the WT strain, although with no statistical significance. The Δ*alb1* mutants, but not the WT strain differentiated sclerotia also on berries and produced higher amount of OTA than the WT strain at both 25 and 30 °C (Table 5).

## 3. Discussion

*A. carbonarius* is a fungus well known as an important source of OTA contamination in food and feed [19]. The fungus belongs to the section *Nigri* of the genus, the so called “black Aspergilli”, which characteristically present dark-brown to black conidia, and hyaline or lightly pigmented hyphae near the apex [20]. DHN melanin is responsible for the dark-brown pigmentation of conidia but it was also proved to be involved in the resistance of conidia to environmental stress and as virulence factor [8,21].

In *A. fumigatus*, a cluster of six genes includes the *alb1* gene encoding a PKS involved in dihydroxynaphtalene (DHN)-melanin biosynthesis [22], which is expressed during conidiation [12]. In addition, deletion of the *alb1* gene determines the albino phenotype of conidia, demonstrating that DHN-melanin is the only pigment responsible for conidia pigmentation in *A. fumigatus* [21]. Although three orthologs of the *A. fumigatus* DHN-melanin biosynthetic genes (*alb1*, *ayg1* and *abr1*) were found in *A. niger*, the deletion of the *alb1* orthologue gene leads to conidia with fawn pigmentation. Hence the residual pigmentation suggests that in addition to DHN melanin, one or more pigments are involved in the blackness of conidia [14].

The gene orthologue to *A. fumigatus alb1* has been detected in *A. carbonarius* and it encodes a type I PKS [16]. The gene is conserved in the ascomycetes and in the *Aspergillus* genus clades largely reflect the sections. The function of the gene was investigated in *A. carbonarius* by comparing three Δ*alb1* mutants with the WT strain. The three Δ*alb1* mutants showed the same behavior and our findings on *A. carbonarius* fit better with genetic data on *A. niger* rather than those on *A. fumigatus*. In fact, in both *A. niger* and *A. carbonarius* the genes close to the *pks* orthologue of *A. fumigatus alb1* gene were not co-expressed and the genes orthologue respectively to *A. fumigatus ayg1* and *abr1* were in a different genetic position, so there is no the evidence for a DHN-melanin biosynthetic gene cluster [14,15,16]. In both species conidia still show a fawn pigmentation following the deletion of the *alb1* gene; hence, in *A. carbonarius* and *A. niger* one or more additional pigment(s) are supposed to be responsible for the black color of conidia and this corroborates the hypothesis that aspergillin, a compound with molecular size of about 20,000 Da, is formed starting from two precursors identified as a hexahydroxyl pentacyclic quinoid (HPQ) and a melanin pigment [5]. A secondary metabolites profile of conidial pigmentation was developed for fungi belonging to *A. niger* group, demonstrating that their conidial pigmentation depends on polyketide-derivative compounds, especially the chemical family of naptho-γ-pyrones, that include aspergillin [23].

The functional characterization of the *alb1* gene was deeply studied in *A. fumigatus* and Δ*alb1* strains produced nearly smooth conidia differently from WT conidia that exhibited echinulate surfaces [8]. Observations through SEM on *A. carbonarius* showed that the surface of conidia of the Δ*alb1* mutants exhibited the same echinulate surface of those of the WT strain (Figure 3), demonstrating that, differently from *A. fumigatus*, the analogue of DHN-melanin is not responsible for conidia ornamentation. In *A. nidulans*, the *wA* gene (orthologue of *alb1*) is regulated by *wetA*, a regulator of spore-specific gene expression [24]. *wetA* together with *brlA* and *abaA* are transcription factors proposed as Central Regulator Pathway (CRP) of conidiogenesis, conserved in the *Aspergillus* genus [25,26]. In a RNA-Seq study on *A. flavus*, *brlA*, *abaA* and *wetA* were shown to be down-regulated in sclerotia [27] and comparable results were obtained by Jin et al. [28] on *A. oryzae*, demonstrating that the down regulation of CRP occurred when *sclR*, a gene involved in the regulation of sclerotia production, was over expressed. Our results showed that the WT strain did not differentiate sclerotia under the adopted growth conditions, whereas all the three Δ*alb1*mutants were able to produce them on all media. The sclerotia pigmentation of Δ*alb1* mutants was not affected, as observed in *Botrytis cinerea* by deleting the same orthologue gene [29]. The relationship between regulatory factors controlling the formation of sclerotia and asexual sporulation is not well understood. However, data described herein showed that in the Δ*alb1* mutants of *A. carbonarius* the conidiogenesis was also significantly reduced as compared to the WT parental strain (Figure 4). These concurrent developmental changes occurring in Δ*alb1*mutants suggest that, probably, the deletion of *alb1* induces the activation of a specific regulation pathway promoting differentially sclerotia production and asexual sporulation processes.

Fungal secondary metabolism and morphological development have been shown to be associated at the genetic level [30,31,32]; for example, in *A. nidulans* and in *Aspergillus* spp. producing aflatoxins, it was demonstrated that a FadA-dependent signal transduction pathway regulate both conidiation and sterigmatocystin-aflatoxins biosynthesis [33]. Another interesting consequence of *alb1* loss in *A. carbonarius* was the statistically significant increase of total OTA production under all tested conditions. In *A. carbonarius*, OTA is differentially partitioned among mycelium, conidia, sclerotia and the medium [34,35]. The amount of OTA accumulated in the WT conidia, ranging from 3.0 to 23.5 pg per 10^2^ conidia, agrees with data by Atoui et al. [35] on the *A. carbonarius* 2Mu134 strain grown on different media. OTA was accumulated in conidia up to three-fold more in the WT strain than in the Δ*alb1* mutants. Conversely, Δ*alb1* mutants were much more active in secreting OTA into the medium as compared to the WT strain and accumulated high amount of OTA in sclerotia. On grape berries artificially inoculated on wounds, Δ*alb1* mutants caused lesions slightly bigger that those caused by the WT strain, demonstrating that the gene is not directly involved in rotting development. They also produced more OTA that the WT strain either at 25 °C or 30 °C confirming the results obtained in vitro on different media.

The biosynthesis of both OTA and DHN-melanin analogues is depending on the PKSs activity, which can be regulated by different environmental factors (e.g., light, pH, nitrogen, and carbon sources) via the activation of specific transcription factors [15]. The existence of common genetic networks between fungal development and secondary metabolism [36] and the different pigmentation of conidia and OTA production and partitioning in different fungal organs showed by the Δ*alb1* mutants and the WT strain suggest that the biosynthesis of both polyketides, OTA, and DHN-melanin, may share common genetic regulation.

In conclusion, this study clarified that the function of the PKS encoding gene *alb1* on conidial pigmentation in *A. carbonarius* is similar to that of the orthologous gene in *A. niger* [14] and different from the genetic mechanism in *A. fumigatus* [8]. The changes observed in Δ*alb1* mutants concerning conidia pigmentation as well as fungal development (conidiogenesis and sclerotia production), OTA production (in vitro and on grape berries) and its partitioning in different fungal organs represent new insights that are worthwhile to be investigated in more detail in *A. carbonarius*.

## 4. Materials and Methods

### 4.1. Strains and Growth Conditions

The *A. carbonarius* wild type (WT) *AC49* strain, stored in the culture collection of the Department of Soil, Plant and Food Sciences of the University of Bari (Italy), and three selected *alb1*-deleted mutants (Δ*alb1-1,* Δ*alb1-2* and Δ*alb1-3*), obtained in the present study, were used. Fungal strains were stored at −80 °C until use and routinely grown on Potato Dextrose Agar (PDA; infusion from 200 g peeled and sliced potatoes kept at 60 °C for 1 h, 20 g dextrose, adjusted at pH 6.5, 20 g agar Oxoid no. 3, per liter) in the dark at 28 ± 1 °C. When required, the medium was supplemented with 100 μg mL^−1^ of hygromycin B (HygB; InvivoGen, San Diego, CA, USA).

*Escherichia coli* DH5α and *A. tumefaciens* AGL-1 strains stored in the culture collection of the Department of Food Biotechnology, Instituto de Agroquimica y Tecnología de Alimentos (IATA-CSIC, Valencia, Spain), were respectively used for the generation of chemically and electrocompetent cells.

### 4.2. Analysis of the A. carbonarius alb1 Gene

The *alb1* gene was identified through blastx analysis on *A. carbonarius* filtered proteins database (http://www.jgi.doe.gov) using the *A. fumigatus alb1* gene (NCBI Accession: AF025541.1) as input sequence. The *A. carbonarius pks* gene (ID: 172075), orthologue of *alb1*, was then used as input for blastx analysis on non-redundant protein sequences of NCBI database (http://www.ncbi.nlm.nih.gov/BLAST/) to identify orthologue genes in other fungi.

The amino acid sequences of the ALB1 acetyl-transferase (AT) domain of 21 *Aspergillus* species and other 15 genera of *Ascomycetes* were obtained by using the Simple Modular Architecture Research Tool (SMART; http://smart.embl-heidelberg.de/). The domain sequences were then aligned through MEGA6 software [37] with the MUSCLE algorithm, and the phylogenetic tree was computed using the neighbor-Joining method [38].

### 4.3. Generation of A. carbonarius Δalb1 Mutants

All primer pairs were designed with the Primer3 software [39]. The amplification of the promoter and the terminator regions (~1.5 kb) from *A. carbonarius* *AC49* genomic DNA (10 ng) was performed using Top-Taq DNA polymerase (Bioron GmbH, Ludwigshafen, Germany), according to manufacturer’s instructions, and the primer pairs ALB1_O1/ALB1_O2 for the promoter and ALB1_A3/ALB1_A4 for the terminator (Table 1). Cycling conditions were: 94 °C for 3 min, 35 cycles of 94 °C for 15 s, 58 °C for 20 s and 72 °C for 2 min and 72 °C for 10 min.

The plasmid pRFHU2-alb1 (Figure 1a) was obtained using the Uracil-Specific Excision Reagent (USER) enzyme (New England Biolabs, Ipswich, MA, USA) by mixing the amplified promoter and terminator fragments with the digested vector pRFHU2 [17] (ratio 30:30:120 ng). Aliquots (1 µL) of the mixture were used for transformation of *E. coli* DH5α chemically-competent cells [17]. After 18 h of incubation at 37 °C on Luria-Bertani (LB) agar medium (bacto tryptone 10 g, yeast extract 5 g, NaCl 5 g, agar 14 g, per liter) supplied with 25 µg mL^−1^ of kanamycin (Invitrogen, Carlsbad, CA, USA), resistant transformants were screened by PCR using the primer pairs RF-5/RF-2 and RF-1/RF-6 (Table 1) and the fusion was confirmed by DNA sequencing.

The plasmid pRFHU2-alb1 was then introduced in electrocompetent *A. tumefaciens* AGL-1 cells using a Gene Pulser apparatus (Bio-Rad, Richmond, CA, USA), generating pulses of up to 2500 V from a 25 μF capacitor. *A. tumefaciens* AGL-1 carrying the plasmid pRFHU2-alb1 was grown at 28 °C for 2 days on LB agar supplemented with kanamycin (50 µg mL^−1^, Invitrogen), rifampicin (20 µg mL^−1^, Sigma-Aldrich, St. Louis, MO, USA) and carbenicillin (75 µg mL^−1^, Sigma-Aldrich, St. Louis, MO, USA). A single colony was used to inoculate LB medium (10 mL) containing the same antibiotics and the culture was incubated for 24 h. AGL-1 cells were centrifuged, washed with the induction medium (IM) [40] and newly suspended (OD600 = 0.15) in the same medium amended with 200 µM acetosyringone (AS; Sigma-Aldrich, St. Louis, MO, USA) (IMAS). Cells were grown at 28 ± 1 °C and 200 rpm until an OD600 of 0.5–0.75 was reached. A 150 µL aliquot of the culture was mixed with an equal volume of a conidial suspension (10^5^ conidia mL^−1^) of *A. carbonarius*, and aliquots (100 µL) of the mixture were spread onto paper filter layered on agar plates containing IMAS. After co-cultivation at 24 ± 1 °C for 40 h, the paper filters were transferred on PDA containing hygromycin B (HygB, 100 µg mL^−1^, InvivoGen), as selective agent for fungal transformants, and cefotaxime (200 µg mL^−1^, Sigma-Aldrich, St. Louis, MO, USA), inhibiting the growth of *A. tumefaciens* cells. *A. carbonarius* *AC49* HygB-resistant colonies appeared after 3–4 days of incubation at 28 ± 1 °C and monosporic cultures were obtained. The genomic DNA was extracted as described by Crespo-Sempere et al. [41]. Transformants were screened for the sequences enclosed between the promoter and the terminator of *alb1* and the *HygB* genes (primer pairs: ALB_1F and hph_1F, ALB_2R and hph_PRO4, respectively), as well as for the deletion of the *alb1* gene (primers ALB1_3F and ALB1_4R) and the insertion of the selection marker *HygB* (primers HMBF1 and HMBR1) (Table 1).

Finally, qPCR was carried out on a sample of 5 selected transformants for assessing the number of T-DNA copies integrated in the genome. The primers ALB1_7F and ALB1_8R (Table 1) were designed in the promoter region of the *alb1* gene close to the selection marker. As reference, the non-ribosomal peptide synthetase (*nrps*) gene (ID: 132610) was used with the primers nrps_1F and nrps_2R (Table 1). qPCR reactions were performed in a final volume of 10 μL, containing 1 × of LightCycler^®^ 480 SYBR Green I Master (Bio-Rad, Hercules, CA, USA), 250 nM of each primer and 10 ng of template DNA. Thermal cycler conditions were: 95 °C for 5 min, followed by 35 cycles at 95 °C for 10 s, 58 °C for 45 s and 72 °C for 10 s. Two technical replicates were performed. All amplifications were carried out in a LightCycler 480 Instrument (Roche Diagnostics, Mannheim, Germany) equipped with LightCycler SW 1.5 software. For each sample, raw data were used to calculate the qPCR efficiency (E) and the quantification cycle (Cq) through LinRegPCR software [42]. The number of T-DNA copies integrated in the genome of each transformant was calculated according to Pfaffl [43].

### 4.4. Morphological Studies and OTA Production

Three *A. carbonarius* Δ*alb1* mutants, Δ*alb1-1,* Δ*alb1-2* and Δ*alb1-3*, were compared with the WT strain for colony growth, conidiogenesis and OTA production. Three different media [PDA; minimal medium (MM; 10 mL solution A (10 g KH_2_PO_4_, 100 mL^−1^ water), 10 mL solution B (20 g NaNO_3_, 5 g KCl, 5 g MgSO_4_·7H_2_O, 0.1 g FeSO_4_, 100 mL^−1^ water), 1 mL of micronutritive solution [44], 20 g glucose, per liter); and coconut-agar medium (CA; 200 g of blended coconut, 18 g agar Oxoid no. 3, per liter)], dispensed in aliquots of 20 mL in Petri dishes of 100 mm diameter, were used in the assays. Mycelial plugs of 4 mm diameter from the edges of actively growing colonies on PDA were used to inoculate three replicated Petri dishes per medium. The orthogonal diameters of developing colonies were measured after 2, 5 and 7 days of incubation at 25 and 30 ± 1 °C in the darkness, which are the optimal conditions for OTA production and vegetative growth in *A. carbonarius*, respectively [45]. Additionally, the production of conidia and OTA was determined in three agar plugs (4 mm diameter) with mycelium and conidia collected from the inner, middle, and outer part of 7-day-old growing colonies [41]. Mature sclerotia were removed before OTA extraction.

### 4.5. OTA Partitioning in WT and Δalb1 Strains

OTA partitioning among conidia, mycelium and the medium was evaluated according to the procedure described by Atoui et al. [31], slightly modified. A sterile cellophane disc was overlayered on CA medium, poured in aliquots (30 mL) in each Petri dish (100 mm diameter), and five replicated dishes were inoculated with mycelial plugs (4 mm diameter) from colonies actively growing on PDA. Colonies were grown up to 10 days at 25 ± 1 °C and at 4, 6, 8 and 10 Days. After Inoculation (DAI) the orthogonal diameters of the developing colonies were measured. Plates were destructively sampled. The entire colony was scraped from the cellophane layer, suspended in 10 mL of sterile water containing 0.01% Tween 20 and shaken vigorously for 5 min. The suspension was filtered through a layer of sterile miracloth, and the filtrate was centrifuged for pelletizing conidia. Mycelium, collected from the miracloth was deprived of sclerotia, when present, and washed three-times with 30 mL of sterile distilled water until complete clearing of conidia. Three 4-mm agar plugs in correspondence of the inner, middle, and outer regions of the growing culture were collected from the same plates for OTA analysis.

### 4.6. Artificial Inoculation on Grape Berries

For each strain, five ripe table-grape berries cv Italia (~35 mm in diameter) collected from two bunches were surface-sterilized with 2% sodium hypochlorite for 1 min, rinsed three times with sterile distilled water and air dried. Conidia, collected by scraping the surface of 7-day-old colonies grown on PDA were suspended in water and adjusted to 10^6^ conidia mL^−1^. Aliquots (10 µL) of the conidial suspension were singly placed on berry skin which was wounded with a needle (3-mm-deep) under the drop. Berries were kept at 100% relative humidity. After 7 DAI at 25 or 30 ± 1 °C in darkness the orthogonal diameters of developing lesion were measured. Five replicated berries inoculated with sterile water were used as control. OTA concentration in berries was assessed as described below.

### 4.7. OTA Extraction and Quantification

OTA was extracted from conidia, mycelium and medium samples adding 5 mL of methanol, vortexing for 2 min, incubating at room temperature for 1 h and filtering through 0.22 µm-pore-size Nuclepore filter (Nucleopore Corp., Pleasanton, CA, USA). All the samples were stored at −20 °C before HPLC analysis. OTA was extracted also from sclerotia when present. All the sclerotia differentiated per plate were separated from the mycelium, weighed, and transferred in a microtube before being crushed with a glass micro-pestle in 5 mL of methanol.

The extraction of OTA from berries was performed according to Visconti et al. [46], slightly modified. Briefly, each berry was suspended in 20 mL of dilution buffer [1% PEG 8000 (Sigma-Aldrich, St. Louis, MO, USA), 0.5% NaHCO3 (Carlo Erba Reagents, Milan, Italy)] and homogenized by using a T-25 high-performance dispersing (Ultra-Turrax, Staufen, Germany). One mL of diluted samples (1:50) was loaded on a Ochratest™ immunoaffinity columns (Vicam, Milford, MA, USA), washed with 5 mL of washing buffer (2% NaCl (Sigma Aldrich, St. Louis, MO, USA), 0.5% NaHCO_3_ (Carlo Erba Reagents, Milan, Italy)) and 5 mL of distilled water, then eluted with 2 mL of methanol.

OTA was quantified through High Performance Liquid Chromatography (HPLC). Briefly, 20 μL of methanol extracts were injected into the chromatographic apparatus made up of an isocratic pump (HP 1100, Agilent Technologies, Santa Clara, CA, USA) equipped with an injection valve (mod. 7125, Rheodyne, Cotati, CA, USA), a fluorometric detector (HP 1100, λ_ex_ = 333 nm, λ_em_ = 460 nm) and a Chemstation Rev A.08.03 data system (Agilent Technologies, Santa Clara, CA, USA). The analytical column was a reversed-phase Discovery C18 (15 cm × 4.6 mm, 5 mm particles) (Supelco, Bellefonte, PA, USA) preceded by a Security Guard (Phenomenex, Torrance, CA, USA). The identification of OTA in extracts was carried out comparing the retention time with that of the OTA standard (Supelco Sigma-Aldrich, St. Louis, MO, USA).

OTA concentration was expressed per mm^2^ of surface of the three sampled agar plugs in the experiment on different media, and the percentage of OTA increase in Δ*alb1* mutants compared to WT was calculated according to the formula: [(OTA_Δ*alb1*_ − OTA_WT_)/OTA_WT_] × 100. In the OTA partitioning experiment, OTA concentration was expressed per g (fresh weight) of mycelium, sclerotia, medium, berry, or number of conidia in the other experiments.

### 4.8. Scanning Electron Microscopy (SEM) Studies

Conidia from 7-day-old colonies on PDA were collected on a 0.22-µm-pore-size Nuclepore filter. The filters were dehydrated, mounted on aluminum stubs, and coated with gold palladium alloy. Conidia were observed under a low-pressure scanning electron microscope Hitachi TM3000 (Hitachi, Tokyo, Japan).

### 4.9. Statistical Analysis

All data were analyzed by ANOVA followed by the Tukey’s honestly significant different test (HSD), using CoStat-software (CoHort Software, Monterey, CA, USA), at the significance levels *p* ≤ 0.05 and *p* ≤ 0.01.

## Figures and Tables

**Table 1:** Primers used in the present work*.*

Primer Name	Primer Sequence (5′-3′)	Target DNA (Organism)
**Amplification of promoter and terminator**		
ALB1_O1	GGTCTTAAUGGAATACCTGGACGCTGTTG	Promoter (*A. carbonarius* WT)
ALB1_O2	GGCATTAAUCGGATCGATTGGGTTGCATT	
ALB1_A3	GGACTTAAUCCAGTGAATGACCGAATGCA	Terminator (*A. carbonarius* WT)
ALB1_A4	GGGTTTAAUAGACTTCGTACGCCACAGAA	
**Screening of transformants**		
RF-5	GTTTGCAGGGCCATAGAC	Promoter (*E. coli* DH5α)
RF-2	TCTCCTTGCATGCACCATTCCTTG	
RF-1	AAATTTTGTGCTCACCGCCTGGAC	Terminator (*E. coli* DH5α)
RF-6	ACGCCAGGGTTTTCCCAGTC	
ALB1_1F	GAACTCACGGCCCTCAAAGA	Promoter (*A. carbonarius* Δ*alb1*)
hph_1F	ACGAGGTCGCCAACATCTTCTTCT	
ALB1_2R	ATTCACCCCGGTTTCCTCAC	Terminator (*A. carbonarius* Δ*alb1*)
hph_PRO4	GCACCAAGCAGCAGATGATA	
HMBF1	CTGTCGAGAAGTTTCTGATCG	Hygromicin (*A. carbonarius* Δ*alb1*)
HMBR1	CTGATAGAGTTGGTCAAGACC	
ALB1_3F	CTTGGTAGGATCCGCGAGAC	*Alb1* (*A. carbonarius* Δ*alb1*)
ALB1_4R	CGGCATCGAAAGCGCAAATA	
**Determination of T-DNA copy number**		
ALB1_7F	ATTTCCGAACGGGGTAACTC	*Alb1* (*A. carbonarius* Δ*alb1*)
ALB1_8R	CAAGGTCTCTTGCAATGCTG	
nrps_1F	GAGCAGCTACCGGAGCTATT	*nrps* (*A. carbonarius* Δ*alb1*)
nrps_2R	GCATCGCATGAGTGAGTTGT	

Underlined: part of the primer useful for the treatment with the USER enzyme mix in the generation of 3′ single stranded overhangs.

**Table 2:** Number of sclerotia observed in the Δ*alb1* mutants as compared to the WT strain.

Strain	Sclerotia (Number ± Standard Error)					
	PDA		MM		CA	
	25 °C	30 °C	25 °C	30 °C	25 °C	30 °C
WT	0 ± 0	0 ± 0	0 ± 0	0 ± 0	0 ± 0	0 ± 0
Δ*alb1-1*	9 ± 1	81 ± 1	4 ± 0	26 ± 2	53 ± 2	61 ± 4
Δ*alb1-2*	23 ± 3	82 ± 1	11 ± 1	25 ± 1	56 ± 5	66 ± 10
Δ*alb1-3*	20 ± 2	108 ± 1	10 ± 1	26 ± 2	44 ± 10	49 ± 4

**Table 3:** Total OTA produced by the Δ*alb1* mutants and the WT strain of *A. carbonarius* in 4-mm-diam agar plugs with mycelium and conidia after 7 days of incubation.

Strain	OTA (ng/mm^2^ ± Standard Error)		
	MM *	PDA	CA
**25 °C**			
WT	11.59 ± 0.67 B	87.89 ± 2.39 B	296.00 ± 22.41 B
Δ*alb1-1*	90.15 ± 12.73 A (677.8) **	124.78 ± 1.19 A (41.9)	492.26 ± 34.18 A (66.3)
Δ*alb1-2*	85.81 ± 7.00 A (640.4)	116.46 ± 3.39 A (32.5)	503.45 ± 13.59 A (70.1)
Δ*alb1-3*	99.21 ± 6.71 A (755.9)	119.69 ± 5.75 A (36.2)	550.03 ± 41.31 A (85.5)
**30 °C**			
WT	12.03 ± 0.55 B	28.80 ± 1.14 B	47.11 ± 2.92 B
Δ*alb1-1*	44.41 ± 5.28 A (269.2)	50.34 ± 1.33 A (70.8)	126.68 ± 5.40 A (168.9)
Δ*alb1-2*	43.97 ± 0.98 A (265.5)	48.35 ± 1.82 A (67.9)	117.97 ± 4.90 A (150.4)
Δ*alb1-3*	39.37 ± 1.19 A (227.2)	43.08 ± 2.64 A (49.6)	112.08 ± 12.43 A (137.9)

Letters refer to comparisons within the same medium for each temperature (Tukey’s test at probability level *p* ≤ 0.01). * MM: Minimal Medium; PDA: Potatoes Dextrose Agar; CA: Coconut Agar. ** In parenthesis: percentage of OTA increase as compared to the wild type calculated with the formula [(OTA_Δ*alb1*_ − OTA_WT_)/OTA_WT_] × 100.

**Table 4:** OTA partitioning in *A. carbonarius* Δ*alb1* mutants and the WT strain grown on CA medium.

Strain	Mycelium (ng mg^−1^)	Conidia (pg 10^−2^ Conidia)	Medium (ng mg^−1^)
**4 DAI**			
WT	3.3 ± 0.8 (a)	21.1 ± 1.8 (a A)	3.8 ± 0.6 (cC)
Δ*alb1-1*	3.0 ± 0.5 (a)	8.9 ± 1.7 (bc B)	9.5 ± 2.1 (ab AB)
Δ*alb1-2*	3.1 ± 0.8 (a)	9.1 ± 1.2 (b AB)	12.8 ± 2.1 (a A)
Δ*alb1-3*	2.6 ± 0.2 (a)	6.1 ± 1.0 (c B)	6.6 ± 0.8 (bc BC)
**6 DAI**			
WT	0.4 ± 0.1 (b A)	23.5 ± 3.9 (a A)	5.1 ± 1.1 (bB)
Δ*alb1-1*	0.9 ± 0.3 (ab A)	12.1 ± 2.5 (ab A)	12.0 ± 1.5 (aAB)
Δ*alb1-2*	1.6 ± 0.4 (a A)	11.3 ± 2.8 (ab A)	13.9 ± 2.2 (a A)
Δ*alb1-3*	1.1 ± 0.5 (ab A)	7.1 ± 2.0 (b A)	12.2 ± 1.7 (a AB)
**8 DAI**			
WT	1.0 ± 0.1 (b A)	3.0 ± 0.5 (a)	9.7 ± 2.4 (a)
Δ*alb1-1*	1.3 ± 0.2 (b A)	1.2 ± 0.2 (a)	13.5 ± 1.5 (a)
Δ*alb1-2*	2.9 ± 0.8 (a A)	1.5 ± 0.2 (a)	12.5 ± 1.6 (a)
Δ*alb1-3*	2.2 ± 0.4 (ab A)	1.3 ± 0.3 (a)	14.5 ± 3.7 (a)
**10 DAI**			
WT	1.0 ± 0.1 (b A)	5.6 ± 0.5 (a A)	8.0 ± 1.0 (b A)
Δ*alb1-1*	2.6 ± 0.7 (ab A)	2.7 ± 0.5 (b B)	13.7 ± 2.5 (a A)
Δ*alb1-2*	1.9 ± 0.3 (ab A)	2.5 ± 0.4 (b B)	12.2 ± 0.7 (ab A)
Δ*alb1-3*	3.3 ± 0.9 (a A)	2.0 ± 0.3 (b B)	11.4 ± 1.0 (ab A)

For each DAI, letters refer to comparisons within the analyzed matrix among strains according to the Tukey’s test at probability levels *p* ≤ 0.05 (lowercase letters) and *p* ≤ 0.01 (uppercase letters).

**Table 5:** Lesion diameter, OTA production and number of sclerotia for the Δ*Alb1* mutants and the WT strain on artificially-inoculated grape berries incubated at two temperatures at 7 DAI.

Strain	25 °C	30 °C
**Lesion diameter (mm)**		
WT	33.2 ± 2.8 a	47.0 ± 1.0 a
Δ*Alb1-1*	35.2 ± 3.0 a	51.3 ± 3.8 a
Δ*Alb1-2*	33.3 ± 0.9 a	52.7 ± 1.4 a
Δ*Alb1-3*	33.3 ± 1.6 a	54.2 ± 5.8 a
**OTA (ng g^−1^)**		
WT	315.1 ± 14.8 a	272.2 ± 26.3 bB
Δ*Alb1-1*	450.4 ± 67.5 a	1380.7 ± 204.8 aAB
Δ*Alb1-2*	515.3 ± 77.3 a	1548.9 ± 187.8 a A
Δ*Alb1-3*	482.8 ± 33.6 a	1929.7 ± 388.3 a A
**Sclerotia (No.)**		
WT	0 ± 0 bC	0 ± 0 bB
Δ*Alb1-1*	2 ± 0 abAB	19 ± 2 aA
Δ*Alb1-2*	1 ± 0 bAB	14 ± 2 aAB
Δ*Alb1-3*	3 ± 0 aA	24 ± 1 aA

Values are the mean values of five replicates. For lesion diameter, OTA, and number of sclerotia, letters refer to comparisons within of the analyzed temperature among strains according to the Tukey’s test at probability levels *p* ≤ 0.05 (lowercase letters) and *p* ≤ 0.01 (uppercase letters).

## Supplementary Materials

The following are available online at http://www.mdpi.com/2072-6651/10/3/120/s1. Figure S1: Grape berries at 7 days after inoculation with Δ*alb1* mutants and WT, Table S1: Determination of the number of T-DNA copies integrated in the genome of *A. carbonarius* transformants, Table S2: Three-way ANOVA on data of conidia and OTA production of *A. carbonarius* Δ*alb1* and WT strains.
