# Supplementary material for: Functional Characterization of the alb1 Orthologue Gene in the Ochratoxigenic Fungus Aspergillus carbonarius (AC49 strain)
Source: Toxins (Basel). 2018 Mar 12;10(3):120. doi: 10.3390/toxins10030120 (PMC5869408; doi:10.3390/toxins10030120)
Supplement: Supplementary file 1 [file toxins-10-00120-s001.pdf]

Supplementary Materials: Functional characterization of the *alb1* orthologue gene in the ochratoxigenic fungus *Aspergilluscarbonarius* (*AC49* strain)

Donato Gerin, Luis González-Candelas, Ana-Rosa Ballester, Stefania Pollastro, Rita Milvia De Miccolis Angelini and Francesco Faretra

Table S1. Determination of the number of T-DNA copies integrated in the genome of *A. carbonarius* transformants.

Strain	Cq_{alb1}	Cq_{NRPS}	ΔCq_{target}	ΔCq_{ref}	GC*
AC49	23.38±0.17	23.29±0.01	-	-	-
Δ*alb1-1*	23.21±0.11	23.20±0.06	0.38	0.35	1.08
Δ*alb1-2*	23.44±0.02	23.41±0.05	0.33	0.31	1.07
Δ*alb1-3*	23.29±0.02	23.29±0.01	0.36	0.33	1.09
Δ*alb1-4*	23.25±0.02	23.40±0.07	0.37	0.31	1.20
Δ*alb1-5*	23.80±0.25	23.84±0.02	0.26	0.23	1.11
Δ*alb1-6*	22.56±0.02	22.52±0.14	0.58	0.54	1.07

*Gene copy number (GC) quantification was conducted by wPCR according to the formula GC = E_{target}^($Cq_{control}$-Cq_{sample})/E_{ref}^($Cq_{control}$-Cq_{sample}). Gene *alb1* was used as target gene for *alb1* transformants, and the *nrps* gene was used as a reference. Wild-type *A. carbonarius* AC49 strain was used as control. All reactions were conducted in duplicate**.**

Table S2. Three-way ANOVA on data of conidia and OTA production of *A. carbonarius* Δ*alb1* and WT strains.

**Source**	**df***	**Conidiogenesis**		**OTA production**	
		F	*p*	F	*p*
Main effects					
Strain (A)	3	122.02	0.00	49.75	0.00
Temperature (B)	1	4.50	0.03	840.68	0.00
Medium (C)	2	3.74	0.03	724.66	0.00
Interactions					
A x B	3	2.86	0.04	12.4	0.00
A x C	6	5.26	0.00	11.83	0.00
B x C	2	5.26	0.01	365.6	0.00
A x B x C	6	4.78	0.00	4.11	0.00

df: degree freedom; F: Fisher ratio; bold $p \leq 0.01$

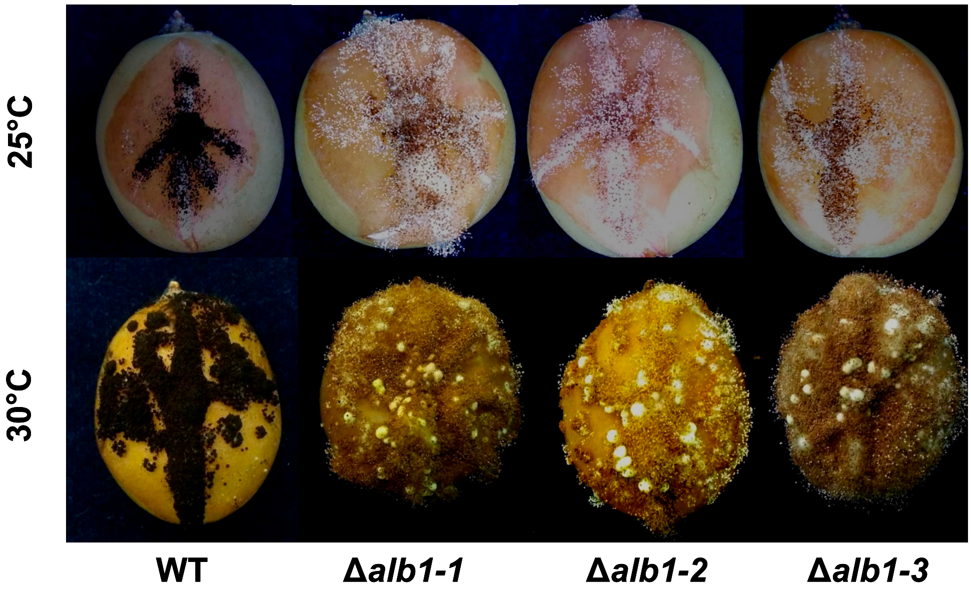

Figure S1. Grape berries at 7 days after inoculation with Δalb1 mutants and WT.
